# Protein subcellular localization prediction for Gram-negative bacteria using amino acid subalphabets and a combination of multiple support vector machines

**DOI:** 10.1186/1471-2105-6-174

**Published:** 2005-07-13

**Authors:** Jiren Wang, Wing-Kin Sung, Arun Krishnan, Kuo-Bin Li

**Affiliations:** 1 Bioinformatics Institute, 30 Biopolis Street, #07-01 Matrix, Singapore 138671; 2 Department of Computer Science, National University of Singapore, 3 Science Drive 2, Singapore 117543

## Abstract

**Background:**

Predicting the subcellular localization of proteins is important for determining the function of proteins. Previous works focused on predicting protein localization in Gram-negative bacteria obtained good results. However, these methods had relatively low accuracies for the localization of extracellular proteins. This paper studies ways to improve the accuracy for predicting extracellular localization in Gram-negative bacteria.

**Results:**

We have developed a system for predicting the subcellular localization of proteins for Gram-negative bacteria based on amino acid subalphabets and a combination of multiple support vector machines. The recall of the extracellular site and overall recall of our predictor reach 86.0% and 89.8%, respectively, in 5-fold cross-validation. To the best of our knowledge, these are the most accurate results for predicting subcellular localization in Gram-negative bacteria.

**Conclusion:**

Clustering 20 amino acids into a few groups by the proposed greedy algorithm provides a new way to extract features from protein sequences to cover more adjacent amino acids and hence reduce the dimensionality of the input vector of protein features. It was observed that a good amino acid grouping leads to an increase in prediction performance. Furthermore, a proper choice of a subset of complementary support vector machines constructed by different features of proteins maximizes the prediction accuracy.

## Background

Subcellular localization is a key functional attribute of a protein. Since cellular functions are often localized in specific compartments, predicting the subcellular localization of unknown proteins may be used to obtain useful information about their functions and to select proteins for further study. Moreover, studying the subcellular localization of proteins is also helpful in understanding disease mechanisms and for developing novel drugs.

As a result of large-scale genome sequencing efforts in recent years, protein data has accumulated in public data banks at an increasing rate. Analyzing protein data to extract useful knowledge is thus essential for projects like automatic annotation. It is desirable to have an automated and reliable system for predicting subcellular localization of proteins from amino acid sequences.

A number of efforts [1-21] have been made to predict protein subcellular localization. Most of these prediction methods can be classified into two categories: one based on the recognition of protein N-terminal sorting signals and the other based on amino acid compositions [22].

Previous works have been focused on protein localization prediction for Gram-negative bacteria. There are five primary localization sites in Gram-negative bacteria, which are the cytoplasm, the extracellular space, the inner membrane, the outer membrane, and the periplasm. PSORT I [23] is the most widely used tool for predicting multiple localizations for Gram-negative bacteria. It uses biological knowledge represented by "if-then" rules for predicting protein localization sites. Most of these rules were derived from experimental observations. However, the PSORT I does not consider the extracellular space site. Additionally, the overall recall for the data set [24] only attains 60.9%.

Gardy et al. [24] presented PSORT-B to improve the prediction performance of PSORT I. PSORT-B combines information of the amino acid composition, similarity to proteins of known localization, presence of a signal peptide, transmembrane alpha-helices and motifs corresponding to specific localizations for a given protein sequence, through a probabilistic approach. It returns a list of five possible localization sites with associated probability scores. It attains an overall recall of 74.8% for the same data set mentioned above.

Recently, Yu et al. [25] proposed a predictive system called CELLO for Gram-negative bacteria by using support vector machines based on n-peptide compositions. They classified 20 amino acids into four groups (charged, polar, aromatic and nonpolar) to reduce the dimensionality of the input vector. Forty SVM classifiers were used to predict the localization sites. Their overall recall was 88.9%. It was a significant improvement over the previous results of PSORT-B. However, the recall for extracelluar proteins was still relatively low at 78.9%.

This paper studies ways to improve the accuracy for predicting extracellular localization in the Gram-negative bacteria. We explored a new way to extract features from protein sequences for protein localization prediction by clustering 20 amino acids into a few groups using a greedy algorithm. Our method for clustering 20 amino acids considers the factors of both amino acids' physical-chemical properties and their contextual correlations. In contrast, the method presented by Yu et al. classifies the 20 amino acids into 4 groups (charged, polar, aromatic and nonpolar) based on physical-chemical properties of amino acids alone. Instead of simply combining multiple SVMs to give a better prediction, we propose a selection score function and a greedy algorithm to select a subset of SVMs to maximize the prediction accuracy.

Based on the proposed approaches, we have developed a system called P-CLASSIFIER for predicting the subcellular localization of Gram-negative bacteria by using a combination of multiple support vector machines. This has resulted in an improvement in the recall for extracellular proteins from 78.9% in CELLO [25] (currently the best predicting system for Gram-negative bacteria) to 86.0% in P-CLASSIFIER. The overall recall of P-CLASSIFIER reaches 89.8%. To the best of our knowledge, these are the most accurate results for predicting protein subcellular localization in Gram-negative bacteria.

## Results

The dataset used in this study is from [24] and was extracted from SWISS-PROT release 40.29 [26]. It contains 1441 proteins of experimentally determined localization, where 1302 proteins are resident at a single localization site and 139 proteins are resident at dual localization sites. Table 1 lists the number of protein sequences from different sites in the data set.

**Table 1:** Number of protein sequences in different sites

**Localization sites**	**No.**
cytoplasmic	248
inner membrane	268
periplasmic	244
outmembrane	352
extracellular	190
cytoplasmic / inner membrane	14
membrane / periplasmic	49
outer membrane / extracellular	76
All sites	1441

The prediction performance of our prediction system is estimated from a 5-fold cross-validation where the given training samples are randomly partitioned into 5 mutually exclusive sets of approximately equal size and approximately equal class distribution.

It is observed that there are some protein sequences in the dataset containing character "X". To avoid possible noise from ambiguous information, the protein entries containing "X" in the protein sequence are excluded in the cross-validation training set, but included in the testing set in this work.

Table 2 shows the prediction recall for single localization. The recall is calculated as TP_x _/ (TP_x _+ FN_x_), where TP_x _and FN_x _represent true positives (number of samples correctly classified as X) and false negatives (number of samples classified as not X that are actually X) over the predictive site X.

**Table 2:** Prediction recall for a single localization.

**Localization**	**Recall (TP_x_/(TP_x_+FN_x_))**
Cytoplasmic	94.8% (235 / 248)
Extracellular	83.2% (158 / 190)
Innermembrane	88.1% (236 / 268)
Outermembrane	93.2% (328 / 352)
Periplasmic	86.9% (212 / 244)
Overall recall	89.8% (1169/1302)

In the dataset, some proteins occur in two different subcellular localizations. Since we are comparing our combined classifier P-CLASSIFIER with the P-SORTB and CELLO classifiers, we followed their method in evaluating the classifier for proteins resident at dual localization sites, where we consider them as predicted correctly if one of their localization sites is predicted correctly. Table 3 shows the prediction recall for dual localizations.

**Table 3:** Prediction recall for dual localizations.

**Localization**	**Recall (TP_x_/(TP_x_+FN_x_))**
Cytoplasmic / innermembrane	92.9% (13/14)
Outermembrane / extracellular	98.9% (75/76)
Periplasmic / innermembrane	75.5% (37/49)
Overall recall	89.9% (125/139)

The Matthews correlation coefficient [27] is used to measure the predictive performance for five predictive sites. The Mattews correlation coefficient (*MCC*) is defined by:



where *TP*_*x*_, *TN*_*x*_, *FP*_*x*_, and *FN*_*x *_are true positives, true negatives (the number of samples correctly predicted as not X that are actually not X), false positives (the number of samples incorrectly predicted as X that are actually not X), and false negatives of localization site X, respectively. *MCC *offers a comprehensive and robust measurement for the predictive performance as this measurement considers both under-and over-predictions. The value of *MCC *equals 1 for a perfect prediction, and 0 for a completely random assignment.

Table 4 lists the performance comparisons among P-CLASSIFIER's (our system), PSORT-B's, and CELLO's [25] systems. As shown in Table 4, the values of *MCC *of all five sites in our system is greater than or equal to the values in CELLO's system, currently the best predicting system for Gram-negative bacteria. Moreover, we increase the recall for the extracellular site from 78.9% in CELLO to 86.0% in P-CLASSIFIER, a significant improvement for the extracellular site on the previous results. The overall recall of P-CLASSIFIER reaches 89.8%, which is better than previous results. To the best of our knowledge, these are the most accurate results for predicting Gram-negative bacteria localization.

**Table 4:** Performance comparisons among P-CLASSIFIER's, PSORT-B's, and CELLO's methods.

	**P-CLASSIFIER**		**CELLO**		**PSORT-B**	
	
**Localization**	**Recall**	***MCC***	**Recall**	***MCC***	**Recall**	***MCC***
Cytoplasmic	94.6%	0.85	90.7%	0.85	69.4%	0.79
Extracellular	86.0%	0.89	78.9%	0.82	70.0%	0.79
Innermembrane	87.1%	0.92	88.4%	0.92	78.7%	0.85
Outermembrane	93.6%	0.90	94.6%	0.90	90.3%	0.93
Periplasmic	85.9%	0.81	86.9%	0.80	57.6%	0.69
Overall recall	89.8%	-	88.9%	-	74.8%	-

## Discussion

To computationally analyse protein data, the representation of protein sequences is an important issue. A good input representation makes it easier for the SVM to identify underlying regularities and therefore is crucial to the success of SVM learning.

In this paper, we encode protein sequences by using the patterns of one amino acid, two adjacent amino acids, three adjacent amino acids, and four adjacent amino acids.

As there are 8000 and 160000 different patterns for the three and four adjacent amino acids cases, clustering 20 amino acids into several groups provides a way to reduce the number of unique patterns since it is difficult to train the SVM with very large number of features such as 160000 for all possible patterns of four adjacent amino acids. Since amino acids in proteins do not contribute to the function of proteins independently and functional patterns in proteins are embedded as sequence correlations, amino acids may not be grouped based on their physical-chemical properties alone [28]. For the prediction task, a good amino acid grouping leads to an improvement in prediction performance.

It is observed that the prediction results from SVMs constructed by different lengths of adjacent amino acid patterns, e.g. the patterns of a single amino acid and amino acid pairs, are complementary. That is, there are some cases where the prediction made by the SVM constructed by patterns of some particular length is correct while the prediction made by the SVM constructed by patterns of another length is incorrect, and vice versa. Therefore, combining complementary results provides a way to improve the prediction accuracy. However, combining all complementary results together may not be a good choice. Therefore, we propose to choose a subset of complementary support vector machines properly that will maximize the prediction accuracy.

After analyzing the predictive results, it is observed that there are some protein sequences that cannot be predicted correctly by any SVM in the combined classifier. It means that these protein sequences cannot be correctly classified by their composition. This is the reason why the recall of some predictive sites in Gram-negative bacteria cannot be further improved.

Since we are comparing our combined classifier P-CLASSIFIER with the P-SORTB and CELLO classifiers, we use the same data set as theirs. We did not check the sequence redundancy in the dataset. As the level of sequence redundancy normally strongly affects prediction accuracy, removing those protein sequences which have high sequence identity (e.g. more than 40%) with each other in the dataset can avoid redundancy and bias.

Instead of giving full credit for dual-localized proteins if either of the sites is predicted correctly, we also evaluate the prediction performance by counting "half" correct when only one of the sites of dual-localized proteins is predicted correctly. Table 5 shows their prediction recalls. The full credit for dual-localized proteins is only given when two possible localization sites with the top two associated probability scores match to actual dual localizations of the protein. The corresponding overall recall for predicting dual localizations only reaches 67.3%. To properly deal with subcellular localizations for proteins resident in several different sites is a challenging problem. The paper [5] addressed the problem of subcellular localizations for proteins resident in several different sites.

**Table 5:** Prediction recall for dual localizations when "half" predictions are only counted as half correct.

**Localization**	**Recall (TP_x_/(TP_x_+FN_x_))**
Cytoplasmic / innermembrane	75.0% (10.5/14)
Outermembrane / extracellular	84.2% (64/76)
Periplasmic / innermembrane	38.8% (19/49)
Overall recall	67.3% (93.5/139)

There are three methods used for cross-validation test: the independent dataset test, n-fold cross-validation test, and the leave one out cross-validation test. Among these methods, the leave one out cross-validation test is the most rigorous and objective [29,42]. However, the leave one out cross validation test is very expensive computationally and is often impractical for large datasets. The n-fold cross-validation test provides a bias-free estimation of the accuracy [30] at much reduced computational cost and is considered as an acceptable test for evaluating predictive performance of an algorithm [31] for large datasets.

## Conclusion

This paper introduces a protein subcellular localization prediction method using amino acid subalphabets and a combination of multiple support vector machines.

The main contributions of our work include: (1) A new way to extract features from protein sequences by clustering 20 amino acids into a few groups using the proposed greedy algorithm to reduce the input dimensionality of support vector machines. Our method for clustering 20 amino acids considers not only the factor of the amino acids' physical-chemical properties but also the factor of their contextual correlations. (2) A selection score function and a greedy algorithm are proposed to select a subset of candidate support vector machines to maximize the cross-validation accuracy instead of simply combining multiple support vector machines to give better prediction. (3) A web-based system has been developed for predicting protein subcellular localization of Gram-negative bacteria. It allows people to submit multiple Gram-negative bacteria protein sequences to perform protein subcellular localization prediction. It is available at [43].

Clustering 20 amino acids into a few groups by our proposed greedy algorithm provides a new way to extract features to cover more adjacent amino acids from protein sequences and reduce the dimensionality of these features. Since amino acids in proteins do not contribute to the function of proteins independently, it may not be a good idea to group amino acids based on their physical-chemical properties alone. For the prediction task, a good amino acid grouping leads to an increase in prediction performance. Furthermore, properly choosing a subset of complementary support vector machines constructed by different features of proteins maximizes the prediction accuracy.

## Methods

### Support vector machines

Support Vector Machines (SVMs) have been widely used in the analysis of biological data [32-34]. SVM is a relatively new family of learning methods and has some theoretical support from statistical learning theory [35,36]. SVM non-linearly maps the input space into a high dimensional feature space, and seeks a hyperplane in this space that separates the positive samples from the negative ones with the largest possible margin and optimizes the trade-off between good classification and large margin. Instead of explicitly mapping the objects to the high dimensional feature space, SVM usually works implicitly in the feature space by only computing the corresponding kernel between any two objects.

Several parameters need to be set during the SVM training phase. These parameters include the regularization parameter, which controls the trade-off between good classification and large margin, the kernel type, and the kernel parameters. These parameters are tuned based on the criteria of cross-validation accuracy. The radial basis function (RBF) kernel is used for all our experiments and the software BSVM [44], a multi-class SVM [37], is used in this work.

### Protein features

The amino acid compositions in the full or partial sequences are considered as global features, which represent the overall similarity among multiple protein sequences. In this paper, the global features are used as the input of the SVMs to predict protein subcellular localization.

#### a. W-gram protein encoding

Two types of features are considered in our work: W-gram and gapped 2-gram. A W-gram is defined as patterns of W (W ≥ 1) consecutive amino acid residues without any gaps and a gapped 2-gram is defined as two amino acid residues with some specified number of gaps in a protein sequence. Here, a gapped 2-gram is also referred to as a 2-gram. The main purpose of introducing the gapped encoding features for 2-gram is to increase the number of 2-gram feature candidates.

For each protein sequence P and each W-gram (or feature) F, let N(P, F) be the number of occurrences of F in the protein sequence P. Further, let T(P, W) be the total number of possible W-grams in P, *length*(P) be the length of P, and G(F) be the specified number of gaps. We have T(P, W) = *length*(P) - W + 1 - G(F), where G(F) = 0 if W ≠ 2 and G(F) ≥ 0 if W = 2. The feature value U(P, F) with respect to the feature F and the sequence P is defined as N(P, F) / T(P, W). For example, suppose P = "LAEVLAAA" and F = "LA" (without any gaps), then the feature value U(P, F) is 2 / (8 - 2 + 1 - 0) = 0.2857, where F = "LA", N(P, F) = 2, *length*(P) = 8, W = 2, G(F) = 0, and T(P, W) = 7. Intuitively, U(P, F) measures the proportional occurrences of F among all possible W-grams in P. This measurement is length independent.

In the W-gram protein encoding method, the total number of different possible features is 20^w^.

#### b. Amino acid subalphabets

It is difficult to train the SVM with very large number of features such as 8000 for 3-gram. To reduce dimensionality, one way is to classify the 20 amino acids into small number of groups based on their physical-chemical properties. All members in the same group can be represented by one symbol. The merged amino acid alphabet has fewer than 20 symbols and is called the amino acid subalphabet, which can be used to re-encode the original protein sequences. The re-encoded protein sequences have fewer features. For example, if the number of symbols in an alphabet is reduced from 20 to 6, the number of 3-gram features is reduced from 4000 (20 × 20 × 20) to 216 (6 × 6 × 6). Reducing the number of features to a manageable size for SVMs can help to improve the predictive performance.

This paper suggests optimizing the grouping by using the proposed greedy algorithm, which considers the factors of both the amino acids' physical-chemical properties and their contextual correlations, instead of using the grouping based on their physical-chemical properties alone. Note that there are an exponential number of ways to group the 20 amino acids. For example, there are 580606446 and 45232115901 ways to divide 20 amino acids into 3 and 4 groups, respectively. The number of subalphabets with m groups (1 ≤ m ≤ 20) for the protein alphabet size of 20, *N(m) *can be calculated by the formula [28] below.



We learn the local optimal grouping based on a greedy algorithm using the SVM classification algorithm to evaluate the fitness of each candidate subalphabet, where the criteria for evaluation is the 5-fold cross-validation accuracy.

#### c. Search for amino acid subalphabets

This section presents our greedy algorithm for finding a good grouping for the amino acids. Given a particular subalphabet encoding schema S, supposing N_g _and T_c _are the predefined number of groups and threshold of cross-validation accuracy, respectively. Further, we assume the parameters of a SVM to evaluate the fitness of a candidate subalphabet are given. These SVM parameters can be set either by the values suggested by the SVM software or by the tuning result of the SVM, which is constructed from features re-encoded by grouping 20 amino acids based on their physical-chemical properties, according to the criteria of cross-validation accuracy. For a particular subalphabet encoding schema S, let the grouping score *h*(S) be the cross-validation accuracy when prediction is done by a SVM using W-gram and the subalphabet scheme S. *h*(S) can be used to measure the goodness of the grouping S.

Table 6 shows an example of clustering 20 amino acids into 4 groups for the 4-gram protein encoding method using the proposed greedy algorithm. The initial node with 4-group assignment is set to {(A, G, I, L, M, P, V), (C, N, Q, S, T), (D, E, K, H, R), (F, W, Y)}, which is based on the physical-chemical property of amino acids. The process for searching for an amino acid subalphabet is iterated until it reaches a local maximal grouping score at 79.0285%, where the final four groups are {(I, L, M, V), (N, S, T), (C, D, E, H, K, Q, R, Y), (A, F, G, P, W)}. Note that some group members in the classified result have the same physical-chemical property of amino acids. For example, the amino acids A, F, G and W in the fourth group (A, F, G, P, W) are all hydrophobic. In particular, the amino acids F and W are aromatic while amino acids A and G are tiny. Further, the hydrophilicity scale indices of A, G, P, and W have approximately the same values in the amino acid index database [38], which suggests that the hydrophilicity of amino acids may be an important property in classifying the 20 amino acids.

**Table 6:** An example of clustering 20 amino acids into 4 groups.

**Searching states**	**Cross-validation accuracy**	**Actions**
(A, G, I, L, M, P, V) (C, N, Q, S, T) (D, E, H, K, R) (F, W, Y)	71.2413%	Move 'G' from group 1 to group 4
(A, I, L, M, P, V) (C, N, Q, S, T) (D, E, H, K, R) (F, G, W, Y)	74.0941%	Move 'A' from group 1 to group 4
(I, L, M, P, V) (C, N, Q, S, T) (D, E, H, K, R) (A, F, G, W, Y)	75.9445%	Move 'P' from group 1 to group 4
(I, L, M, V) (C, N, Q, S, T) (D, E, H, K, R) (A, F, G, P, W, Y)	77.5636%	Move 'C' from group 2 to group 3
(I, L, M, V) (N, Q, S, T) (C, D, E, H, K, R) (A, F, G, P, W, Y)	78.4888%	Move 'Q' from group 2 to group 3
(I, L, M, V) (N, S, T) (C, D, E, H, K, Q, R) (A, F, G, P, W, Y)	78.9514%	Move 'Y' from group 4 to group 3
(I, L, M, V) (N, S, T) (C, D, E, H, K, Q, R, Y) (A, F, G, P, W)	79.0285%	Reach local maximal grouping score and stop.

The proposed greedy algorithm to search for amino acid subalphabets is described in Table 7. The greedy local search [39] has been used for learning the subalphabets. In the search tree [39], every node represents an amino acid subalphabet encoding schema. The child nodes of a node are subalphabets encoding schemata, which are generated by moving every group member to each other group if the number of members in this group is greater than one.

**Table 7:** Algorithm for amino acid subalphabets searching

1	current_node ← the initial group assignment by dividing the 20 amino acids into N_g _groups.
2	REPEAT
3	best_node ← current_node
4	REPEAT
5	current_node ← best_node
6	generate all child nodes of the current node in the search tree.
7	best_node ← the child node with the highest *h*-value among all child nodes of the current node.
8	UNTIL *h*(best_node) <*h*(current_node)
9	IF *h*(current_node) < T_c _THEN
10	current_node ← randomly re-generate initial group assignment
11	ENDIF
12	UNTIL *h*(current_node) ≥ T_c_

This algorithm is composed of the following four steps. First, the 20 amino acids are initially divided into N_g _groups either randomly with approximately the same size or based on some physical-chemical properties of the 20 amino acids. Amino acids in the same group are denoted by one symbol in a subalphabet. Suppose the current subalphabet encoding schema is represented by current node, its grouping score is calculated where the grouping score is the cross-validation accuracy when prediction is done by a SVM using W-gram and this subalphabet scheme.

Second, all child nodes of the current node are generated. If there is only one member in some group, this member cannot move to any other group. Otherwise, the total number of groups will be less than N_g_. There are at most 20 × (N_g _- 1) possible child nodes in the searching space since there are 20 amino acids and each amino acid can only move to at most (N_g _- 1) other groups. If the highest grouping score among the child nodes is greater than the grouping score of the current node, this child node will become the current node.

Third, the above process for searching the child node with the highest grouping score among all child nodes will be repeated until the grouping scores of all child nodes are less than the grouping score of the current node.

Fourth, if the grouping score in the final current node is greater than T_c_, the N_g _groups in the current node will become the accepted merged subalphabets. Otherwise, we randomly re-generate the current node and repeat the Steps 2 to 4 above.

The training sequences are divided into two parts: One part is used for choosing the subalphabet while the other is used for evaluating the performance of a subalphabet.

The greedy algorithm is applied to reduce the number of W-gram features. In particular, for 3-gram, we classify the 20 amino acids into 6, 7, and 8 groups. For 4-gram, we classify the 20 amino acids into 4 groups. The number of features is *m*^W^, where *m *is the number of groups and W is the number of protein peptides in W-gram encoding methods. For example, the number of features is 6 × 6 × 6 = 216 for 6 groups in 3-gram encoding method.

### Multiple SVMs

Due to the nature of the multi-class classification, it may not be easy to obtain a single SVM that can return high accuracies for the subcellular localization prediction. Therefore, multiple SVMs are trained from different features and their results are combined using voting.

Currently most of the existing protein subcellular localization prediction systems using SVMs only use the features generated from 1-gram or 2-gram protein encoding methods. For example, the extracted features of amino acid compositions [2] and features of amino acid pair and gapped amino acid pair compositions [40] can be considered as the features generated from the 1-gram and 2-gram encoding methods, respectively.

As many functional patterns in proteins are embedded as sequence correlations, it is expected that more information will be included by combining classifiers constructed from features generated by 1-gram, 2-gram, 3-gram, and 4-gram protein encoding methods, instead of just using the classifiers constructed from 1-gram and 2-gram encoding methods since more adjacent amino acid residues will be considered.

In this paper, the following four types of features are extracted from protein sequences. The first type is the 1-gram encoding feature, which includes amino acid compositions and the partitioned amino acid compositions, where the protein sequence is partitioned into P parts with approximately the same length. The total number of these features is 20 × P. In this work, P is set from 2 to 6. The second one is 2-gram encoding feature, which includes amino acid pair and gapped amino acid pair compositions, where the number of features is 400 (20 × 20) and the number of gaps is set from 1 to 2. The purpose of introducing the gapped encoding features only for 2-gram is to increase the number of 2-gram feature candidates. The third one is the 3-gram encoding feature, where the 20 amino acids are divided into 6, 7, and 8 groups whose numbers of features are 216 (6 × 6 × 6), 343 (7 × 7 × 7), and 512 (8 × 8 × 8), respectively. The last one is the 4-gram encoding method, where the 20 amino acids are divided into 4 groups, whose number of features is 256 (4 × 4 × 4 × 4).

### Feature selection

We apply the wrapper approach [41] in the backward elimination version to select the feature subset for our SVM classifiers and use 5-fold cross-validation accuracy as the criteria for evaluation.

Let SVM_a _and SVM_b _be the SVM classifiers using all features and features selected by the wrapper approach, respectively. Although the prediction accuracy of SVM_b _is improved, the prediction results from SVM_a _and SVM_b _are different. There are some cases where the prediction made by SVM_a _is correct while the prediction made by SVM_b _is not correct, and vice versa. Therefore, both SVM_a _and SVM_b _can be considered as candidates to build the final combined classifier.

### SVM subset selection

Different SVMs give different predictions. One way to combine their predictions is by voting. That is, each protein sequence is assigned to a class with the most votes. For cases where two or more classes get the most votes, we assign these cases to the predictive results by one of the SVMs, which gets the most number of correct predictions for all these cases.

Suppose S is a set of protein sequences, N is the number of candidate SVMs, M = {SVM_1_, SVM_2_, ..., SVM_N_} is the set of candidate SVMs defined previously, V_1_(S, M) is the number of correct predictions classified by M with only one class corresponding to the most vote, and V_2_(S, M) is the number of the correct predictions by the assigned SVM when two or more classes correspond to the most vote. The selection score function V(S, M) is defined as V_1_(S, M) + V_2_(S, M) and is used to select a subset of all candidate SVMs to form a combined classifier, which maximizes the cross-validation accuracy. The proposed greedy algorithm to select a subset of M is described in Table 8.

**Table 8:** Algorithm for SVM subset selection

1	Let M = {SVM_1_, SVM_2_, ..., SVM_N_} be the set of candidate SVMs
2	Let Score_max _= V(S, M) and Set_max _= M
3	FOR i = N-1 to 1
4	V_max _= max{V(S, M - {SVM_r_}) | SVM_r _∈ M, 1 ≤ r ≤ N }
5	IF V(S, M - {SVM_j_}) == V_max _(1 ≤ j ≤ N) THEN
6	M = M - {SVM_j_}
7	ENDIF
8	IF V_max _≥ Score_max _THEN
9	Score_max _= V_max_
10	Set_max _= M
11	ENDIF
12	END FOR

This greedy algorithm consists of the following two steps. First, set M = {SVM_1_, SVM_2_, ..., SVM_N_}, Score_max _= V(S, M), Set_max _= M, and i = N - 1. Second, for every member SVM_r _∈ M (1 ≤ r ≤ N), remove SVM_r _from M and calculate the value of its corresponding selection score function V(S, M - {SVM_r_}) (1 ≤ r ≤ N). Suppose for some SVM_j _(1 ≤ j ≤ N), V(S, M - {SVM_j_) is equal to V_max_, the maximal value of all V(S, M - {SVM_r_) (1 ≤ r ≤ N), then update the following: M = M - {SVM_j_, Score_max _= V_max_, Set_max _= M, and i = i - 1.The process for removing some SVM_p _(1 ≤ p ≤ N) will continue until i = 1, that is, only one SVM is left. Then Set_max _is selected to be the combined classifier.

We can use the prediction results of four-fifth training protein sequences to select a subset of SVMs and use the prediction results of the rest of one-fifth training protein sequences to evaluate the performance of the result of the SVM subset selection.

In this work, 15 SVMs are selected and combined to form the final classifier. Table 9 shows the encoding methods of input vectors in the fifteen selected SVMs. Rows 12, 13, and 14 represent 3 different merged subalphabets, which are {(A, F, G, P, W), (C, D, E, H, K, Q, R, Y), (N, S, T), (I, L, M, V)}, {(A, C, M, P, V), (F, I, L, W), (D, E, H, Q, R), (G, K, N, S, T, Y)}, and {(A, G, P, Q, Y), (C, D, E, H, K, M, R), (N, S, T), (F, I, L, M, V)}, respectively. Rows 4, 7 and 15 represent the same encoding method as the rows 3, 6 and 14 but with feature selection.

**Table 9:** The encoding methods of input vectors in the fifteen selected SVMs.

**No.**	**Encoding methods of input vectors**
1	1-gram with 2 partitioned parts
2	1-gram with 3 partitioned parts
3	1-gram with 4 partitioned parts
4	1-gram with 4 partitioned parts (apply feature selection to No. 3)
5	1-gram with 6 partitioned parts
6	2-gram without any gaps
7	2-gram without any gaps (apply feature selection to No. 6)
8	2-gram with one gap
9	3-gram with 6 merged groups
10	3-gram with 7 merged groups
11	3-gram with 8 merged groups
12	4-gram with 4 merged groups
13	4-gram with 4 merged groups
14	4-gram with 4 merged groups
15	4-gram with 4 merged groups (apply feature selection to No. 14)

We have conducted some experiments on constructing SVMs by using 5-gram encoding method. Preliminary experimental results show that the cross-validation accuracies predicted by SVM constructed by 3-gram, 4-gram, and 5-gram encoding methods are not satisfactory when the number of groups is less than 6, 4, and 4, respectively. When we increase the number of groups to 4 for 5-gram, the time required to train the corresponding SVM and calculate the 5-fold cross validation accuracy is relatively slow as the number of features reaches 1024 (4 × 4 × 4 × 4 × 4). Therefore, only 1-gram, 2-gram, 3-gram, and 4-gram encoding methods are considered in this paper. Furthermore, the 20 amino acids are classified into 6, 7, and 8 groups for 3-gram and 4 groups for 4-gram encoding methods, respectively.

Since there are too many zero elements in the encoding results, 2-gram, 3-gram, and 4-gram protein's encoding methods are not applied to those cases where the protein sequences are partitioned into P (P > 1) parts with approximately same length.

## Authors' contributions

JW developed the methods, built the system and drafted the manuscript. WS, AK and KL participated in system design, provided valuable comments, and helped to draft the manuscript.
